# Supplementary material for: MetaRibo-Seq measures translation in microbiomes
Source: Nat Commun. 2020 Jun 29;11:3268. doi: 10.1038/s41467-020-17081-z (PMC7324362; doi:10.1038/s41467-020-17081-z)
Supplement: Supplementary file 10 — Supplementary Data 7 [file 41467_2020_17081_MOESM10_ESM.zip › File2/Confidence_High_Taxonomy/93720_out.krona.html]

Javascript must be enabled to view this page.

members
magnitude
magnitudeUnassigned
count
unassigned
taxon
rank

93720\_out

5

superkingdom
2
5

976
5
phylum

200643
5
class

5
171549
order

family
5
815

5
816
genus

species

SRS147272\_contig\_number\_1347SRS147377\_contig\_number\_10178
28116
2

371601
1

SRS1041143\_contig\_number\_3774
species

162156
1

SRS048060\_contig\_number\_11085
species

species

SRS046712\_contig\_number\_111
818
1
